# Biological effects of cinnamaldehyde in animal cancer models: a systematic review and meta-analysis

**DOI:** 10.3389/fphar.2025.1557088

**Published:** 2025-05-16

**Authors:** Dan Luo, Xu Luo, Jinghui Xie, Keying Ye, Xiangjin Wang, Xiyue Hu, Chenhao Liu, Yi Liu

**Affiliations:** ^1^ College of Basic Medicine, Chengdu University of Traditional Chinese Medicine, Chengdu, China; ^2^ Hospital of Chengdu University of Traditional Chinese Medicine, Chengdu, China; ^3^ School of Sports Medicine and Health, Chengdu Sports University, Chengdu, China

**Keywords:** mechanism, cinnamaldehyde, cancer, meta-analysis, animal experiment

## Abstract

**Background:**

Cinnamaldehyde (CA), a naturally occurring aromatic aldehyde from cinnamon bark, has been investigated for its biological activity in laboratory settings. However, its α,β-unsaturated aldehyde structure designates it as a pan-assay interference compound (PAINS), which can produce non-specific effects through chemical reactivity—particularly in vitro—raising concerns about the validity and interpretation of its reported anti-tumor activity.

**Objective:**

To systematically review and synthesize existing animal studies that examine the biological effects of CA on tumor growth, while critically evaluating the strength, limitations, and plausibility of the evidence, especially in light of CA’s PAINS-related characteristics.

**Methods:**

A systematic literature search was conducted across eight electronic databases to identify relevant animal studies assessing the effects of CA on tumor progression. Study quality was evaluated using the Systematic Review Centre for Laboratory Animal Experimentation (SYRCLE) risk of bias tool. Quantitative synthesis was performed using Review Manager (RevMan) 5.3. *In vitro* studies were excluded due to concerns regarding non-specific activity and limited translatability.

**Results:**

Sixteen studies encompassing 19 independent experiments and 302 animals were included. Pooled results indicated that CA administration was associated with reductions tumor volume and tumor weight in animal models. However, no improvement in survival was observed, and CA-treated animals showed a modest decrease in body weight. Additionally, reduced expression of proliferating cell nuclear antigen (PCNA), hypoxia-inducible factor (HIF), vascular endothelial growth factor (VEGF), and microvessel density was reported. Despite these findings, the absence of controls for. Non-specific reactivity makes it difficult to distinguish true pharmacological effects from general cytotoxic or chemical stress responses.

**Conclusion:**

While CA has demonstrated anti-tumor effects in animal models, these observations should be interpreted with caution. Its classification as a PAINS compound, coupled with a lack of mechanistic specificity, appropriate controls, and clinical validation, limits the reliability and translational relevance of the existing data. The observed outcomes are more likely reflective of non-specific chemical activity rather than targeted therapeutic action. Future research should prioritize rigorous mechanistic validation, use of non-reactive analogs, and comprehensive toxicity profiling before considering any clinical applicability.

## 1 Introduction

The global cancer burden continues to pose a major public health challenge. According to the latest GLOBOCAN 2022 epidemiological data, there are approximately 20 million new cases annually, with 9.7 million deaths worldwide (Bray et al., 2024). In recent decades, treatment modalities have expanded beyond conventional approaches, transitioning from traditional treatments such as surgical resection such as surgical resection, chemotherapy, and radiotherapy, to modern strategies, including molecular-targeted therapy, gene therapy, and immunotherapy (Liu B. et al., 2024). However, despite these advances, treatment outcomes remain limited due to the complexity of the Tumor Microenvironment (TME) and resistance mechanisms, resulting in therapeutic resistance, limited long-term efficacy, and minimal improvements in overall survival rate (Emens et al., 2017; Zheng et al., 2018).

These limitations have sparked growing interest in plant-derived natural products as potential sources for novel compounds with biological activity (Liu W. et al., 2024). Natural compounds have contributed significantly to modern drug discovery, particularly in oncology, due to their structural diversity, multi-target mechanisms (Newman and Cragg, 2016). Several plant-derived compounds have successfully entered clinical use, with vinblastine, camptothecin, and paclitaxel becoming components of current cancer therapies (Bae et al., 2015; Buyel, 2018). These successes highlight the potential value of investigating natural products for the development of new therapeutic approaches (Zhang et al., 2024; Liu et al., 2020).

Among the many natural compounds studied in laboratory settings, cinnamaldehyde (CA) has been investigated in numerous animal experiments. CA (C9H8O; (E)-3-phenylprop-2-enal; CAS: 14371-10-9, Figure 1), a naturally occurring aromatic aldehyde, is primarily isolated from two species of Cinnamomum: Cinnamomum verum (Ceylon cinnamon) and Cinnamomum cassia (Chinese cinnamon) (Cruz-Tirado et al., 2023). CA has been classified as Generally Recognized as Safe (GRAS) by both the United States Food and Drug Administration and the Flavor and Extract Manufacturers Association, with additional approval from the European Food Safety Authority for use as a food additive (Friedman, 2017).

**FIGURE 1:**
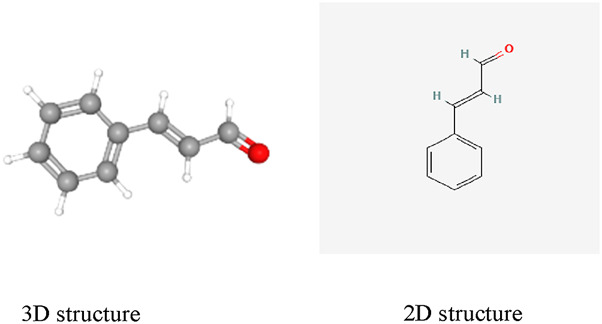
The chemical structure of Cinnamaldehyde.

Beyond its conventional applications, laboratory studies have investigated various biological properties of CA, including antioxidant (Das et al., 2022), antimicrobial (Doyle and Stephens, 2019) and antidiabetic properties (Zhu et al., 2017). Animal experiments have reported associations between CA exposure and various cellular changes in cancer models (Peng et al., 2024), including effects on signaling pathways such as nuclear factor kappa B (NF-κB), mitogen-activated protein kinase (MAPK), and nuclear factor erythroid 2-related factor 2 (Nrf2) pathways. Some researchers have proposed that CA may modulate key tumor-associated molecules, such as p53, Bcl-2 family proteins, cyclin D1, and vascular endothelial growth factor (VEGF) (Banerjee and Banerjee, 2023).

However, when evaluating the reported anticancer effects of CA, it is essential to recognize the hierarchy and limitations of existing evidence. Current evidence primarily falls into three levels: (1) *in vitro* cellular experiments, which provide preliminary mechanistic insights but often fail to reflect the complex *in vivo* environment; (2) animal model studies, which offer data more representative of integral biological systems but are limited by species differences and dosage translation issues; and (3) human clinical studies, which are completely lacking for CA’s anticancer properties.

Importantly, CA’s α,β-unsaturated aldehyde structure classifies it as a pan-assay interference compound (PAINS) (Magalhães et al., 2021), capable of forming non-specific adducts with nucleophilic residues in biological molecules. This reactivity complicates the interpretation of mechanistic studies, as observed effects may arise from chemical artifacts rather than specific target engagement. Therefore, this meta-analysis was conducted to comprehensively assess CA’s effects across various cancer models based on available animal studies, while critically considering the potential limitations related to its PAINS classification and the overall quality of the current evidence.

## 2 Methods

This systematic review and meta-analysis was conducted in accordance with the Preferred Reporting Items for Systematic Reviews and Meta-Analyses (PRISMA) guidelines (Liberati et al., 2009). The protocol was registered with the International Prospective Register of Systematic Reviews (registration ID: CRD42024568407).

### 2.1 Search stratege

Two researchers (DL and XL) independently performed a comprehensive search across eight databases: PubMed, Embase, Web of Science, Science Direct, Google Scholar, China National Knowledge Infrastructure (CNKI), Chongqing VIP China Science Technology Journal Database (VIP), and Wanfang Data Knowledge Service Platform (Wanfang). The search included studies from each database’s inception to November 2024, with no restrictions on language or publication year. Search terms included a combination of MeSH and free-text keywords. For PubMed, the strategy used: (“Cinnamaldehyde”[Mesh]) AND (“Neoplasms”[Mesh]) AND (“Animal Experimentation”[Mesh] OR “Models, Animal”[Mesh]). Full search strategies for all databases are detailed in Supplementary Material 1. Reference lists of included articles and relevant reviews were also manually screened for additional eligible studies. Disagreements regarding inclusion were resolved through discussion or, if needed, by a third reviewer (YL).

### 2.2 Study selection and eligibility criteria

Studies were selected based on the population, intervention, comparison, and outcomes (PICO) framework. The inclusion criteria were as follows: (1) *In vivo* cancer models using mice or rats, regardless of tumor type; (2) Use of CA as the main intervention; (3) Inclusion of a control group (e.g., untreated or vehicle-treated animals); and (4) Reported and quantifiable outcome measures related to tumor growth. Preference was given to studies that included non-reactive or structurally related analogs (e.g., cinnamic acid, hydrocinnamaldehyde) as negative controls, to help differentiate specific biological effects from non-specific chemical activity. Studies were excluded based on the following criteria: (1) *In vitro* studies, clinical trials, reviews, conference abstracts, or other meta-analyses; (2) Animal models with comorbid conditions; (3) Studies lacking essential data or inaccessible full text; (4) And duplicate or overlapping publications. Two reviewers independently screened titles, abstracts, and full texts. Discrepancies were resolved through consensus or adjudication by a third reviewer.

### 2.3 Data extraction

Literature management was conducted using EndNote X9. Two researchers independently extracted the following data: publication year, authors, animal species, age, sex, tumor type and cell line, CA dosage, route and duration of administration, sample sizes, and outcome measures. Quantitative data presented only in a graphical form were extracted using GetData Graph Digitizer 2.20. When multiple dose groups were reported, the highest dose group was used for analysis. Data from the final observation point were used to maximize consistency. Standard errors of the mean (SEM) were converted to standard deviations (SD) using the formula: SD = SEM × √n (Lee et al., 2015), where n is the sample size. All discrepancies were resolved by discussion or by contacting the original authors where possible.

### 2.4 Risk of bias assessment

The methodological quality of the included studies was evaluated using the Systematic Review Centre for Laboratory Animal Experimentation (SYRCLE) risk of bias tool for animal studies (Hooijmans et al., 2014). This tool assesses biases across six domains: (1) selection bias, evaluating sequence generation, baseline comparability, and allocation concealment; (2) performance bias, assessing randomization of housing and personnel blinding protocols; (3) detection bias, considering randomization of outcome assessment and blinding of outcome assessors; (4) attrition bias, regarding the reporting of incomplete outcome data; (5) reporting bias, examining selective reporting of outcomes; and (6) other sources of bias that may impact study validity. Each domain was rated as “low risk,” “high risk,” or “unclear.” Two reviewers conducted the assessment independently, and discrepancies were resolved through discussion.

### 2.5 Data synthesis and analysis

Meta-analyses were performed using the RevMan 5.3. Due to the substantial heterogeneity across preclinical animal studies, a random-effects model was employed. For continuous variables, standardized mean differences (SMDs) and 95% confidence intervals (CIs) were calculated. Statistical significance was defined at *P* < 0.05. Heterogeneity was assessed using the *I* ^*2*^ statistic, where values exceeding 50% indicated the need for a random-effects model, and values below 50% suggested the use of a fixed-effects model (Wong et al., 2018). Based on the Cochrane Handbook for Systematic Reviews of Interventions (Higgins et al., 2003), an *I* ^2^ value below 50% was considered to represent low heterogeneity, 50%–90% moderate heterogeneity, and greater than 90% high heterogeneity. Where appropriate, subgroup analyses were conducted based on species, tumor type, dose, route of administration, and treatment duration. Dose-response and time-course analyses focused on tumor volume and weight changes in studies with statistically significant results (P < 0.05).

## 3 Results

### 3.1 Study selection

**FIGURE 2:**
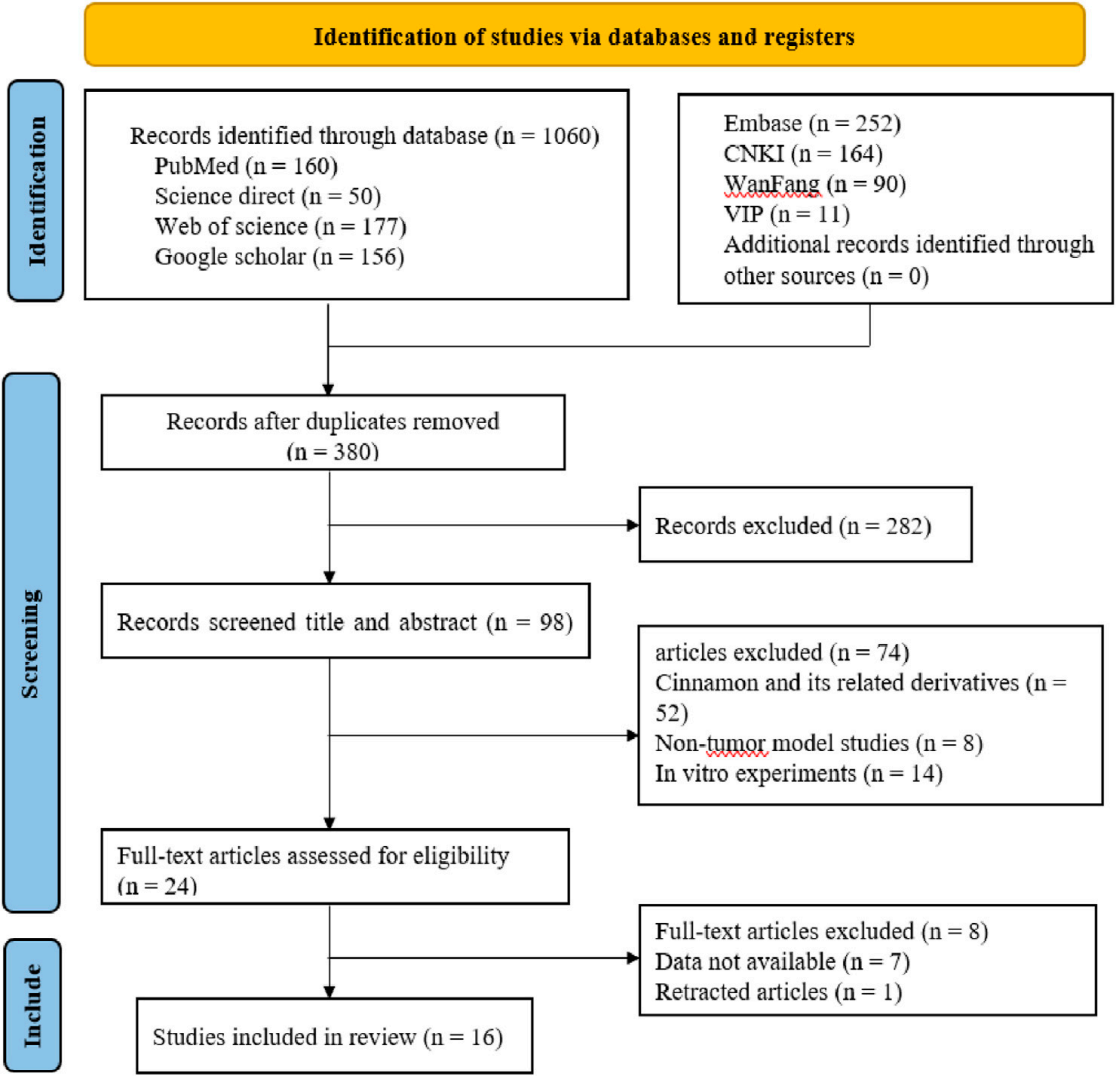
Flow diagram for the process of included studies identification.

A systematic literature search across eight electronic databases yielded a total of 1,060 records: PubMed (n = 160), Science Direct (n = 50), Web of Science (n = 177), Google Scholar (n = 156), Embase (n = 252), CNKI (n = 164), Wanfang Data (n = 90), and VIP (n = 11). After removing duplicates using the EndNote and conducting manual screening, 380 articles remained for further evaluation. Subsequently, 282 records were excluded based on title, abstract, or study type (e.g., reviews, meta-analyses, conference abstracts, case reports, and studies not focused on cancer models). Of the remaining 98 articles, 74 were excluded due to irrelevance or ineligibility-specifically, studies on CA derivatives (n = 52), non-cancer models (n = 8), and in vitro-only investigations (n = 14). Full-text screening of 24 articles led to the exclusion of 8 additional studies due to insufficient data extraction (n = 7) or article retraction (n = 1). Ultimately, 16 studies (Bae et al., 2015; Huang et al., 2006; Yin et al., 2017; Chiang et al., 2019; Chu et al., 2022; Wu et al., 2019; Wang et al., 2022; Imai et al., 2002; Zhang et al., 2023; Chen et al., 2021; Cabello et al., 2009; Zhou et al., 2014; Tian et al., 2017; Huang, 2021; Li et al., 2014; Gopalakrishnan et al., 2023) met all inclusion criteria and were included in the final analysis. A summary of the study selection process is presented in Figure 2.

### 3.2 Characteristics of included studies

**FIGURE 3:**
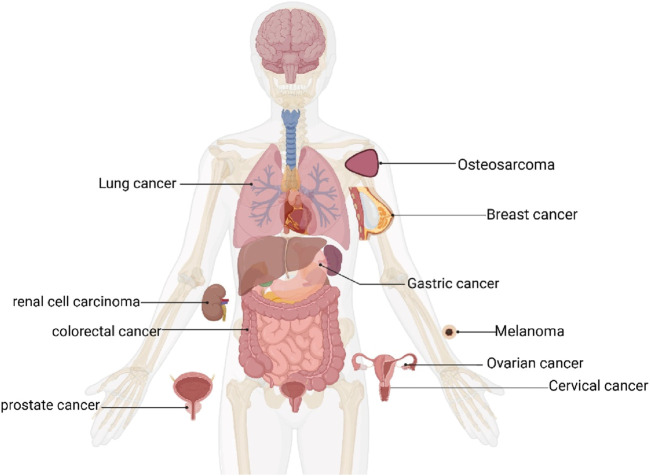
Anatomical illustration showing the distribution of cancer types in the included studies.

This final analysis included 16 publications, comprising 19 independent *in vivo* experiments. These studies collectively involved 302 tumor-bearing animals, with 137 assigned to CA treatment groups and 165 to control groups. All studies utilized either mice or rat models. Among them, 11 studies used BALB/c and its variants (114/302, 37.75%), four studies used CB6F1 mice (96/302, 31.79%), 1 study used Mice (40/302, 13.25%), 1 study used Nude mice (10/302, 3.31%), 1 study used SCID mice (22/302, 7.28%), and 1 study used Wistar rats (20/302, 6.62%). Of the 19 studies, 9 used female animals, 6 used male animals, 2 used both male and female animals, and 2 did not specify the animal sex. Thirteen studies mentioned the age of the animals. However, only 3 studies provided information on animal weight, while the majority (16 studies) did not describe animal weight. Regarding tumor models, 4 studies employed drug-induced tumor models, whereas 15 studies utilized xenograft tumor models (solid tumors). As shown in Figure 3, the cancer types included lung cancer (6 studies), melanoma (3 studies), colorectal cancer (2 studies), osteosarcoma (2 studies), gastric cancer, cervical cancer, breast cancer, ovarian cancer, renal cell carcinoma, and prostate cancer (one study each). Across all studies, the minimum treatment duration was 2 weeks, with a maximum of 26 weeks. The minimum CA dosage was 2 mg/kg (administered every 3 days), whereas the maximum dosage was 240 mg/kg/d. For outcome measures, 8 studies recorded tumor weight, 9 studies recorded tumor volume, and 11 studies recorded body weight changes. Among these, 2 studies observed all three indicators simultaneously, 6 studies observed two indicators, and 13 studies observed only a single indicator.

Among the 20 included studies, Sigma-Aldrich (St. Louis, MO, USA) was the predominant reagent source (6 studies), followed by Wako Pure Chemicals (Osaka, Japan; 4 studies). Six studies used CA sourced from Chinese manufacturers, while two did not specify the supplier. Reagent purity was reported in 13 studies (ranging from 95% to 99%): 5 studies ≥95%, 6 studies ≥98%, and 2 studies ≥99%. Four studies did not report purity. Additionally, quality control methods were documented in only 8 studies, which included capillary gas chromatography (n = 4), high-performance liquid chromatography (n = 2), standard gas chromatography (n = 1), and reversed-phase HPLC (n = 1). The remaining 12 studies did not report any analytical confirmation of CA purity or identity. Detailed experimental and chemical characteristics are summarized in Tables 1, 2.

**TABLE 1:** Information of CA of each study.

Study (years)	Source	Purity (%)	Quality control reported
Huang et al., 2006	Sinopharm chemical Reagent Co., Ltd	>98%	NR
Yin et al., 2017	Sigma–Aldrich (St. Louis, MO, USA)	≥95%	NR
Chiang et al., 2019	Sigma-Aldrich (St. Louis, MO, USA)	≥95%	NR
Chu et al., 2022	NR	NR	NR
Wu et al., 2019	China National Institute for the Control of Pharmaceutical and Biological Products	99%	NR
Wang et al., 2022	Aladdin, Shanghai, China	≥99.5%	GC
Bae et al., 2015	Sigma–Aldrich (St. Louis, MO, USA)	≥95%	NR
Imai et al., 2002	Wako Pure Chemicals (Osaka, Japan)	≥98%	Capillary GC
Imai et al., 2002	Wako Pure Chemicals (Osaka, Japan)	≥98%	Capillary GC
Imai et al., 2002	Wako Pure Chemicals (Osaka, Japan)	≥98%	Capillary GC
Imai et al., 2002	Wako Pure Chemicals (Osaka, Japan)	≥98%	Capillary GC
Zhang et al., 2023	ShanghaiyuanyeBio-TechnologyCo., Ltd	≥98%	HPLC
Chen et al., 2021	Shanghai BS Bio-Tech Co., Ltd (Shanghai, China)	99.41%	HPLC
Cabello et al., 2009	Sigma Chemical Co, St. Louis, MO	NR	NR
Zhou et al., 2014	NR	NR	NR
Tian et al., 2017	Sigma–Aldrich (St. Louis, MO, USA)	≥95%	NR
Huang. 2021	chengdu Herbpurify Co., Ltd (chengdu, China)	NR	RP-HPLC
Li et al., 2014	Hubei Yuancheng Pharmaceutical Co., Ltd (Hubei, China)	NR	NR
Gopalakrishnan et al., 2023	Sigma–Aldrich (St. Louis, MO, USA)	≥95%	NR

Notes: Capillary GC, capillary gas chromatography; GC, gas chromatography; HPLC, high performance liquid chromatography; RP-HPLC, Reversed-Phase High Performance Liquid Chromatography; NR, not report.

**TABLE 2:** Basic characteristics of the included studies.

Study (years)	Sample				Number (T/C)	Cell strain	Concentration of cells (cell/mouse)	Tumer model	Type of tumor	Intervention		Control	Outcomes
	Species	Sex	Age	Weight						Nature/dosage	Administration/Douration/Frequency		
Huang et al., 2006	BALB	Half male and female	NR	19–22 g	8/8	SGC-7901	1 × 10^8^/mL	XM(S) - Xenograft model (Solid tumor)	Gastric cancer	CA suspended in DMSO/100 mg/kg	By intraperitoneal injection/21day/qd	Saline	①
Yin et al., 2017	Mice	Female	NR	18–20 g	20/20	U14	5 × 10^6^/mL	XM(S) - Xenograft model (Solid tumor)	Cervical cancer	CA suspended in sterile water/240 mg/kg	By intragastric/14d/qd	Placebo	①③
Chiang et al., 2019	BALB/c nude mice	Female	5 weeks	NR	3/3	MDA-MB-231-GFP cells	2 × 10^7^/mL	XM(S) - Xenograft model (Solid tumor)	Breast cancer	CA mixed into the regular diet/100 mg/kg	By free access to the diets/8 weeks/qd	Vsfatin	①②
Chu et al., 2022	BALB/c AnN.CgFoxnnu/Crl Narl mice	Male	5 weeks	NR	5/5	143B cells	1 × 10^7^/mL	XM(S) - Xenograft model (Solid tumor)	Osteosarcoma	CA suspended in sterile water/2 mg/kg	By intragastric/30 days/Once every 3 days	Placebo	③
Wu et al., 2019	BALB/c/nu/nu nude mice	Half male and female	6–8 weeks	NR	5/5	HCT116 cells	1 × 10^6^	XM(S) - Xenograft model (Solid tumor)	Colorectal cancer	CA suspended in DMSO/50 mg/kg	By intraperitoneal injection/3w/qd	Oxaliplatin	①②
Wang et al., 2022	Nude mice	Female	6 weeks	NR	5/5	A2780 cells	5 × 10^6^	XM(S) - Xenograft model (Solid tumor)	Ovarian cancer	CA suspended in DMSO/100 mg/kg	By intraperitoneal injection/3 weeks/Once every 3 days​	Phosphate buffer saline	①
Bae et al., 2015	BALB/c	Male	6 weeks	NR	3/3	renal adenocarcinoma cell line	5 × 10^6^	XM(S) - Xenograft model (Solid tumor)	Renal cell carcinoma	CA suspended in DMSO/10 mg/kg	By intraperitoneal injection/2 weeks/qd	Saline	②③
Imai et al., 2002	CB6F1-TgHras2 (rasH2) mice	Male	NR	NR	8/16	The 4-(Methylnitrosamino)- 1-(3-pyridyl)-1-butanone (NNK)	3mg/mouse	Drug-induced model	Lung cancer	CA mixed into the regular diet/0.5%	By free access to the diets/26 weeks/qd	Placebo	③
Imai et al., 2002	CB6F1-TgHras2 (rasH2) mice	Female	NR	NR	8/16	The 4-(Methylnitrosamino)- 1-(3-pyridyl)-1-butanone (NNK)	3mg/mouse	Drug-induced model	Lung cancer	CA mixed into the regular diet/0.5%	By free access to the diets/26 weeks/qd	Placebo	③
Imai et al., 2002	CB6F1-nonTgHras2 (non-Tg)	Male	NR	NR	8/16	The 4-(Methylnitrosamino)- 1-(3-pyridyl)-1-butanone (NNK)	3mg/mouse	Drug-induced model	Lung cancer	CA mixed into the regular diet/0.5%	By free access to the diets/26 weeks/qd	Placebo	③
Imai et al., 2002	CB6F1-nonTgHras2 (non-Tg)	Female	NR	NR	8/16	The 4-(Methylnitrosamino)- 1-(3-pyridyl)-1-butanone (NNK)	3mg/mouse	Drug-induced model	Lung cancer	CA mixed into the regular diet/0.5%	By free access to the diets/26 weeks/qd	Placebo	③
Zhang et al., 2023	BALB/c-nude mice	Female	6 weeks	15 g	5/5	HCT-116 cells	1 × 10^7^	XM(S) - Xenograft model (Solid tumor)	Colorectal cancer	NR/80 mg/kg	By subcutaneous injection/2 weeks/qd	Saline	①②③
Chen et al., 2021	BALB/c nude mice	Female	5 weeks	NR	5/5	A549 cells	5 × 10^6^	XM(S) - Xenograft model (Solid tumor)	Lung cancer	CA dissolved in dimethyl sulfoxide/100 mg/kg	By intraperitoneal injection/27days/qd	PBS	①②③
Cabello et al., 2009	SCID mous	NR	6–8 weeks	NR	12/10	Human A375 melanoma cells	1 × 10^7^	XM(S) - Xenograft model (Solid tumor)	Melanoma	CA suspended in 0.5% methylcellulose/PBS/120 mg/kg	By intragastric/30days/qd	0.5% methylcellulose/PBS	②
Zhou et al., 2014	BALB/c null	Female	2–4 weeks	NR	6/4	The melanoma cells	1 × 10^7^	XM(S) - Xenograft model (Solid tumor)	Melanoma	CA solution, 2 mg/kg	Subcutaneous Injection	Saline	②
Tian et al., 2017	BALB/c nude mice	Male	4–6 weeks	NR	5/5	NCI-H460 cells	NR	XM(S) - Xenograft model (Solid tumor)	Lung cancer	CA suspended in DMSO/100 mg/kg	By intraperitoneal injection/3w/qd	PBS	①
Huang. 2021	BALB	NR	4 weeks	NR	3/3	143B cells	2 × 107	XM(S) - Xenograft model (Solid tumor)	Osteosarcoma	CA suspended in DMSO/75 mg/kg	By intragastric/19days/qd	Sodium Carboxymethyl Cellulose	②③
Li et al., 2014	BALB/c null	Female	2–4 weeks	NR	10/10	The melanoma cells	1 × 10^5^	XM(S) - Xenograft model (Solid tumor)	Melanoma	CA solution, 2 mg/kg	By intraperitoneal injection/3w/qd	Saline	②
Gopalakrishnan et al., 2023	Wistar/NIN rats	Male	3 months	200–250 g	10/10	N-methyl-N-nitrosourea (MNU)	50 mg/kg	Drug-induced model	Prostate cancer	CA mixed into the regular diet/150 mg/kg	By free access to the diets/16w/qd	Placebo	③

Note: ①, Tumor weight; ②, Tumor, volume; ③, body weight; NR, not report.

### 3.3 Quality of the included studies

**FIGURE 4:**
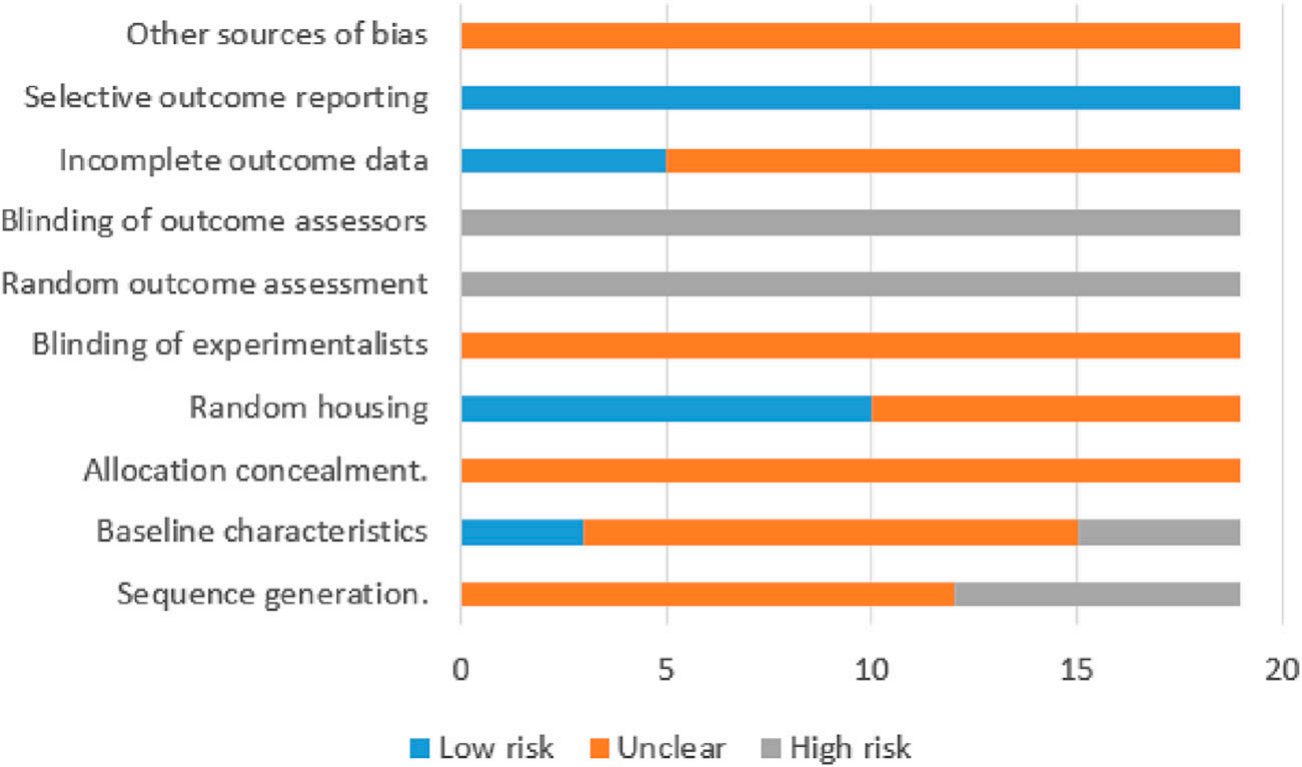
Risk of bias graph.

Risk of bias was assessed for all 19 experiments using the SYRCLE risk of bias tool, as illustrated in Figure 4. The studies received scores ranging from 1 to 3. Nine articles (Bae et al., 2015; Huang et al., 2006; Wu et al., 2019; Wang et al., 2022; Zhang et al., 2023; Chen et al., 2021; Zhou et al., 2014; Huang, 2021; Li et al., 2014) received 1 point, 3 articles (Chu et al., 2022; Cabello et al., 2009; Tian et al., 2017) received 2 points, and 4 articles (Yin et al., 2017; Chiang et al., 2019; Imai et al., 2002; Gopalakrishnan et al., 2023), containing 7 independent experiments, received 3 points. Of the total 19 evaluated experiments, 12 studies mentioned random assignment but lacked details, while 7 made no reference to randomization, indicating potential selection bias. Only 3 experiments reported comparable group characteristics at baseline, which was evaluated as low risk of bias. Notably, no study indicated whether the allocation among groups was adequately concealed, and this was assessed as unclear risk assessment. Ten experiments used identical housing conditions and environmental parameters for experimental animals, which was assessed as low risk of bias. All experiments were classified as high-risk due to the absence of information regarding the random selection of animals for outcome assessment. Notably, none of the studies documented blinding of personnel or outcome assessors, constituting a high risk for performance and detection bias. Five studies provide explained regarding whether missing data affected the authenticity of the final results, which were evaluated as low risk of bias. Although all included studies comprehensively reported predetermined outcomes (low risk of bias), the evaluation of other potential sources of bias remains unclear across studies. A detailed quality assessment of the included studies is presented in Table 3.

**TABLE 3:** Risk of bias summary.

Author(year)	A	B	C	D	E	F	G	H	I	J	Total
Huang et al., 2006	?	?	?	+	?	-	-	?	+	?	1
Yin et al., 2017	?	?	?	+	?	-	-	+	+	?	3
Chiang et al., 2019	?	+	?	+	?	-	-	?	+	?	3
Chu et al., 2022	?	+	?	?	?	-	-	?	+	?	2
Wu et al., 2019	?	?	?	?	?	-	-	?	+	?	1
Wang et al., 2022	-	?	?	?	?	-	-	?	+	?	1
Bae et al., 2015	-	?	?	?	?	-	-	?	+	?	1
Imai et al., 2002	-	-	?	+	?	-	-	+	+	?	3
Imai et al., 2002	-	-	?	+	?	-	-	+	+	?	3
Imai et al., 2002	-	-	?	+	?	-	-	+	+	?	3
Imai et al., 2002	-	-	?	+	?	-	-	+	+	?	3
Zhang et al., 2023	?	?	?	?	?	-	-	?	+	?	1
Chen et al., 2021	?	?	?	?	?	-	-	?	+	?	1
Cabello et al., 2009	-	?	?	+	?	-	-	?	+	?	2
Zhou et al., 2014	?	?	?	?	?	-	-	?	+	?	1
Tian et al., 2017	?	?	?	+	?	-	-	?	+	?	2
Huang. 2021	?	?	?	?	?	-	-	?	+	?	1
Li et al., 2014	?	?	?	?	?	-	-	?	+	?	1
Gopalakrishnan et al., 2023	?	+	?	+	?	-	-	?	+	?	3

Note: A, Sequence generation. B, Baseline characteristics. C, Allocation concealment. D, Random housing. E, Blinding of experimentalists. F, Random outcome assessment. G, Blinding of outcome assessors. H, Incomplete outcome data. I, Selective outcome reporting. J, other sources of bias.

### 3.4 Primary outcomes

#### 3.4.1 Tumor weight

**FIGURE 5:**
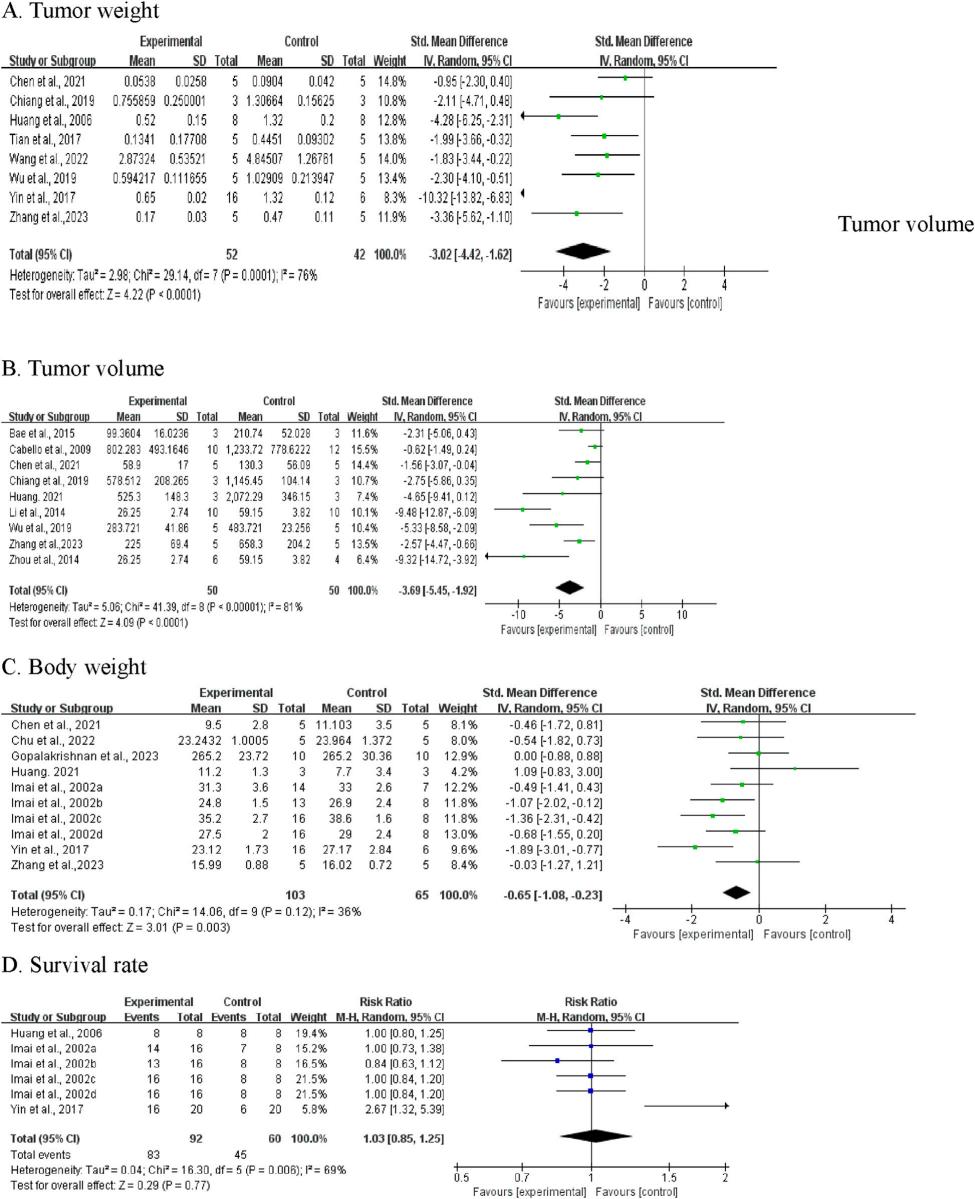
Meta‐analysis results of BBR on primary outcomes: **(A)** tumor weight; **(B)** tumor volume; **(C)** Body weight; **(D)** Survival rate.

Of the 19 included studies, 8 studies documented the effects of CA on tumor weight (Bae et al., 2015; Huang et al., 2006; Yin et al., 2017; Chiang et al., 2019; Wu et al., 2019; Wang et al., 2022; Zhang et al., 2023; Tian et al., 2017) (Figure 5A). Given the significant heterogeneity (*I* ^*2*^ = 76%; *P* = 0.0001), we employed a random-effect model. The pooled effect demonstrated that CA administration was associated with reduced tumor weight compared to the control group (SMD = −3.02; 95% CI [-4.42, −1.62]).

Sensitivity analyses were conducted to assess the stability of these studies. The results revealed no significant changes after sequential exclusion of individual studies. However, it is noteworthy that the exclusion of Yin et al. (2017) resulted in an adjusted SMD of −2.24 (95% CI [-3.08, −1.41]), with heterogeneity decreasing from 76% to 32%, suggesting that this study may be the primary source of heterogeneity.

Given the considerable heterogeneity, subgroup analyses were performed based on drug dosage, duration, and tumor type. Owing to the limited number of published articles, some subgroups contained only one article. As shown in Table 4, subgroup analyses revealed that tumor type was a potential influencing factor, with CA reducing tumor weight in gastric, cervical, colorectal, ovarian, and lung cancers, while showing limited effects on breast cancer tumor weight, as no difference was observed (SMD: -2.11, 95% CI: −4.71, 0.48, *P* = 0.11). The dosage and treatment duration of CA did not appear to be major factors explaining the heterogeneity, as the *I* ^*2*^ values showed no significant differences between the subgroups.

**TABLE 4:** Subgroup analysis for the effects of CA on tumor weight.

Study characteristics	No. of studies	Test for heterogeneity			Test for effect	
		Chi^2^ test	*H-P-value*	*I* ^2^ (%)	SMD (95% CI)	*E-P-value*
**Total**	8	29.14	0.0001	76	−3.02 (−4.42, −1.62)	<0.00001
1.Dose						
Between-subgroup heterogeneity		0.89	0.35	0		
≥100 mg/kg	5	27.91	<0.0001	86	−3.59 (−5.97, −1.2)	0.003
<100 mg/kg	3	1.17	0.56	0	−2.33 (−3.39, −1.27)	<0.0001
3.Duration						
Between-subgroup heterogeneity		1.72	0.19	41.8		
≥21 days	6	7.63	0.18	34	−2.11 (−3.00, −1.22)	<0.00001
<21d	2	10.74	0.0001	91	−6.71 (−13.52, 0.11)	0.05
4.Tumor type						
Between-subgroup heterogeneity		27.72	<0.0001	82		
gastric cancer	1	NA	NA	NA	−4.28 (−6.25, −2.31)	<0.0001
Cervical cancer	1	NA	NA	NA	−10.32 (−13.82, −6.83)	<0.00001
breast cancer	1	NA	NA	NA	−2.11 (−4.71, 0.48)	0.11
colorectal cancer	2	0.52	0.47	0	−2.71 (−4.12, −1.31)	0.0002
Ovarian cancer	1	NA	NA	NA	−1.83 (−3.44, −0.22)	0.03
lung cancer	2	0.9	0.34	0	−1.36 (−2.41, −0.31)	0.01

Note: SMD: standardized mean difference, negative values indicate reduction in tumor weight, larger absolute SMD, values indicate stronger intervention effects; CI: confidence interval, all 95% CIs, are represented using square brackets (.); *H-P*-value: Heterogeneity *P*-value; *E-P*-value: Effect *P*-value; *P* < 0.05 was considered statistically significant; NA: not applicable, indicates subgroups with only one study where heterogeneity statistics could not be calculated.

#### 3.4.2 Tumor volume

Nine studies measuring tumor volume were included (Bae et al., 2015; Chiang et al., 2019; Wu et al., 2019; Zhang et al., 2023; Chen et al., 2021; Cabello et al., 2009; Zhou et al., 2014; Huang, 2021; Li et al., 2014). Pooled analysis showed that CA treatment was associated with decreased tumor volume versus control groups (SMD = −3.69, 95% CI: -5.45 to −1.92, *P* < 0.0001) (Figure 5B). Given the substantial heterogeneity (*I* ^*2*^ = 81%, *P* < 0.00001), we performed subgroup analyses by experimental species, CA dosage, duration, routes, and tumor types. As shown in Table 5, subgroup analyses revealed that CA dosage was the main source of heterogeneity. The high-dose subgroup (≥100 mg/kg) showed reduced heterogeneity (*I* ^*2*^ = 19%). Subgroup analyses based on treatment duration, administration routes, and tumor types showed no heterogeneity (*I* ^*2*^ = 0%), suggesting these factors did not contribute to the heterogeneity. Sensitivity analysis through one-by-one study removal confirmed the robustness of our findings on CA’s tumor-suppressive effects.

**TABLE 5:** Subgroup analysis for the effects of CA on tumor volume.

Study characteristics	No. of studies	Test for heterogeneity			Test for effect	
		Chi^2^ test	*H-P-value*	*I* ^2^ (%)	SMD (95% CI)	*E-P-value*
Total	9	41.39	<0.00001	81	−3.69 (−5.45, −1.92)	<0.0001
1.Model species						
Between-subgroup heterogeneity		11.33	0.0008	91.2		
Mice	8	25.51	0.0006	73	4.25 (−6.17, −2.32)	<0.0001
Rat	1	NA	NA	NA	−0.62 (−1.49, 0.24)	0.16
2.Dose						
Between-subgroup heterogeneity		9.3	0.002	89.2		
≥100 mg/kg	3	2.46	0.29	19	−1.06 (−1.97, −0.16)	0.02
<100 mg/kg	6	17.87	0.03	72	−5.24 (−7.76, −2.71)	<0.0001
3.Duration						
Between-subgroup heterogeneity		0.32	0.57	0		
≥21 days	5	33.89	<0.00001	88	−4.04 (−6.81, −1.26)	0.004
<21d	4	2.83	0.42	0	−3.15 (−4.50, −1.80)	<0.00001
4.Administration						
Between-subgroup heterogeneity		2.39	0.50	0		
intraperitoneal injection	3	4.27	0.12	53	−2.70 (−4.74, −0.65)	0.01
intragastric	2	2.65	0.1	62	−1.93 (−5.62, 1.76)	0.31
free access to the diets	1	NA	NA	NA	−2.75 (−5.86, 0.35)	0.08
subcutaneous injection	3	15.26	0.0005	87	−6.83 (−12.18, −1.48)	0.01
5.tumor type						
Between-subgroup heterogeneity		4.04	0.54	0		
breast cancer	1	NA	NA	NA	−2.75 (−5.86, 0.35)	0.08
colorectal cancer	2	2.08	0.15	52	−3.62 (−6.26, −0.99)	0.007
renal cell carcinoma	1	NA	NA	NA	−2.31 (−5.06, 0.43)	0.1
melanoma	3	33.14	<0.00001	94	−6.22 (−13.30, 0.86)	0.09
lung cancer	1	NA	NA	NA	−1.56 (−3.07, −0.04)	0.04
Osteosarcoma	1	NA	NA	NA	−4.65 (−9.41, 0.12)	0.06

Note: SMD: standardized mean difference, negative values indicate reduction in tumor volume, larger absolute SMD, values suggest stronger intervention effects; CI: confidence interval, all 95% CIs, are represented using square brackets (.); *H-P*-value: Heterogeneity *P*-value; *E-P*-value: Effect *P*-value; *P* < 0.05 was considered statistically significant; NA: not applicable, indicates subgroups with only one study where heterogeneity statistics could not be calculated.

It’s worth noting that for some tumors and doses, the SMD results appear significant, but the corresponding *P*-values are non-significant (e.g., breast cancer SMD = −2.75, *P* = 0.08) or borderline significant (0.05 < *P* < 0.10, as seen in melanoma and osteosarcoma). This pattern mainly occurs in subgroups with only one study or small sample sizes. Due to the lack of replicate studies, when the SMD is large, the confidence interval is wide, resulting in non-significant *P*-values despite potentially meaningful effect sizes. *P*-values between 0.05 and 0.10 suggest a trend toward significance that may become statistically significant with larger sample sizes. These early findings show therapeutic promise but require further investigation. Future studies focusing on specific tumor types and doses are needed to better assess the consistency and significance of effects in these subgroups.

#### 3.4.3 Body weight

Body weight was reported in seven studies (ten independent experiments), with 103 animals in treatment groups and 65 in control groups (Yin et al., 2017; Chu et al., 2022; Imai et al., 2002; Zhang et al., 2023; Chen et al., 2021; Huang, 2021; Gopalakrishnan et al., 2023). The analysis revealed that the groups receiving CA demonstrated a decrease in body weight compared to the control groups (SMD = −0.65, 95% CI [-1.08, −0.23], *P* = 0.003) (Figure 5C). The test showed low heterogeneity (*I* ^*2*^ = 36%), therefore a fixed-effects model was adopted.

#### 3.4.4 Survival rate

In terms of survival, data from three studies (6 experiments) were synthesized, including 92 mice in the experimental group and 60 in the control group (Huang et al., 2006; Yin et al., 2017; Imai et al., 2002). Analysis showed that RR = 1.03 (95% CI: 0.85-1.25; *P* = 0.77). Given the moderate heterogeneity among studies (*I* ^*2*^ = 69%, *P* = 0.006), a random-effects model was utilized (Figure 5D). The results revealed no significant difference in survival rates between CA-treated and control animals.

### 3.5 Secondary outcomes

#### 3.5.1 PCNA protein expression

**FIGURE 6:**
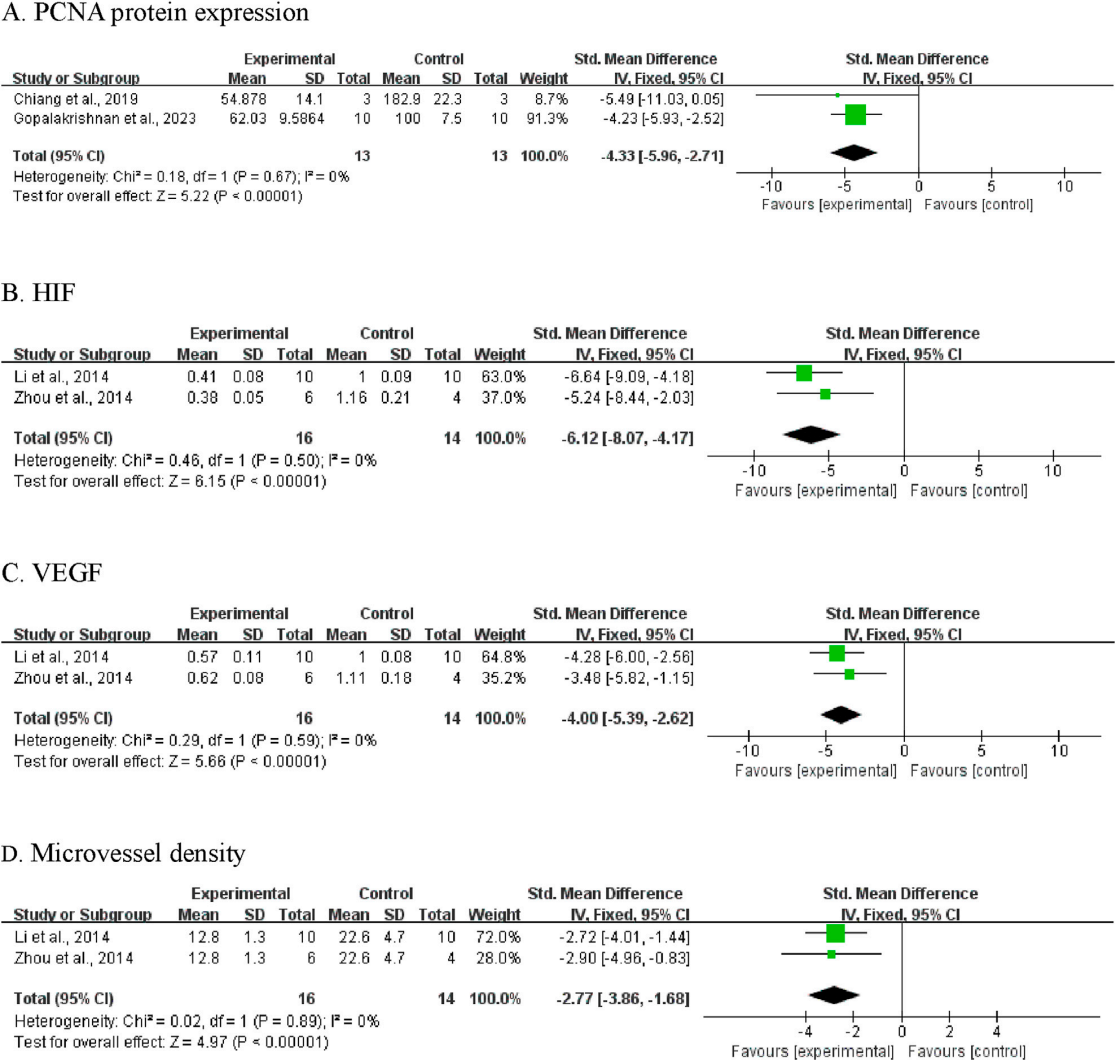
Meta‐analysis results of BBR on secondary outcomes: **(A)** PCNA protein expression; **(B)** HIF; **(C)** VEGF; **(D)** microvessel density.

Proliferating Cell Nuclear Antigen (PCNA) expression was assessed in two independent studies involving 26 mice (13 per group) (Cabello et al., 2009; Gopalakrishnan et al., 2023). Meta-analysis revealed that CA-treated groups exhibited significantly lower PCNA protein levels compared to control groups (SMD = −4.33; 95% CI [-5.96, −2.71]; *P* < 0.00001) (Figure 6A). No heterogeneity was observed (*I* ^*2*^ = 0%, *P* = 0.67); therefore, a fixed-effects model was applied.

#### 3.5.2 Hypoxia-inducible factor (HIF)

Two studies reported data on HIF protein expression (Zhou et al., 2014; Li et al., 2014). The pooled analysis showed significantly decreased HIF levels in CA-treated animals compared to controls (SMD = −6.12, 95% CI [-8.07, −4.17]; *P* < 0.00001; Figure 6B). As no heterogeneity was detected (*I* ^*2*^ = 0%), a fixed-effects model was used.

#### 3.5.3 Vascular endothelial growth factor (VEGF)

Analysis of VEGF expression, reported in two studies (Zhou et al., 2014; Li et al., 2014), demonstrated lower VEGF protein levels in the CA-treated groups (SMD = −4.00, 95% CI [-5.39, −2.62]; *P* < 0.00001; Figure 6C). A fixed-effects model was employed due to low heterogeneity (*I* ^*2*^ = 0%, *P* = 0.59).

#### 3.5.4 Microvessel density

Two studies assessed tumor microvessel density (Zhou et al., 2014; Li et al., 2014). Results indicated that CA treatment was associated with a significant reduction in microvessel density, suggesting suppression of tumor associated angiogenesis (SMD = −2.77, 95% CI [-3.86, −1.68]; *P* < 0.00001; Figure 6D). Heterogeneity was not detected (*I* ^*2*^ = 0%, *P* = 0.89).

### 3.6 Publication bias assessment

**FIGURE 7:**
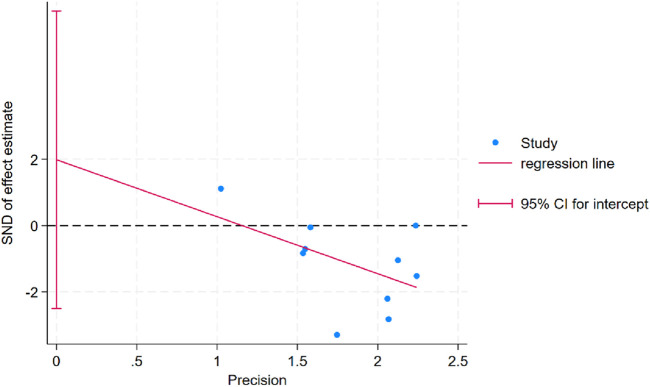
Egger’s publication bias plot for body weight.

Egger’s test was conducted for body weight outcomes and indicated no evidence of publication bias (*P*Egger = 0.337; Figure 7). For other outcome variables, publication bias could not be assessed due to the limited number of studies (n < 10), which does not meet the minimum threshold recommended by the Cochrane Handbook for meta-analytic bias evaluation.

### 3.7 Dose and duration analysis

**FIGURE 8:**
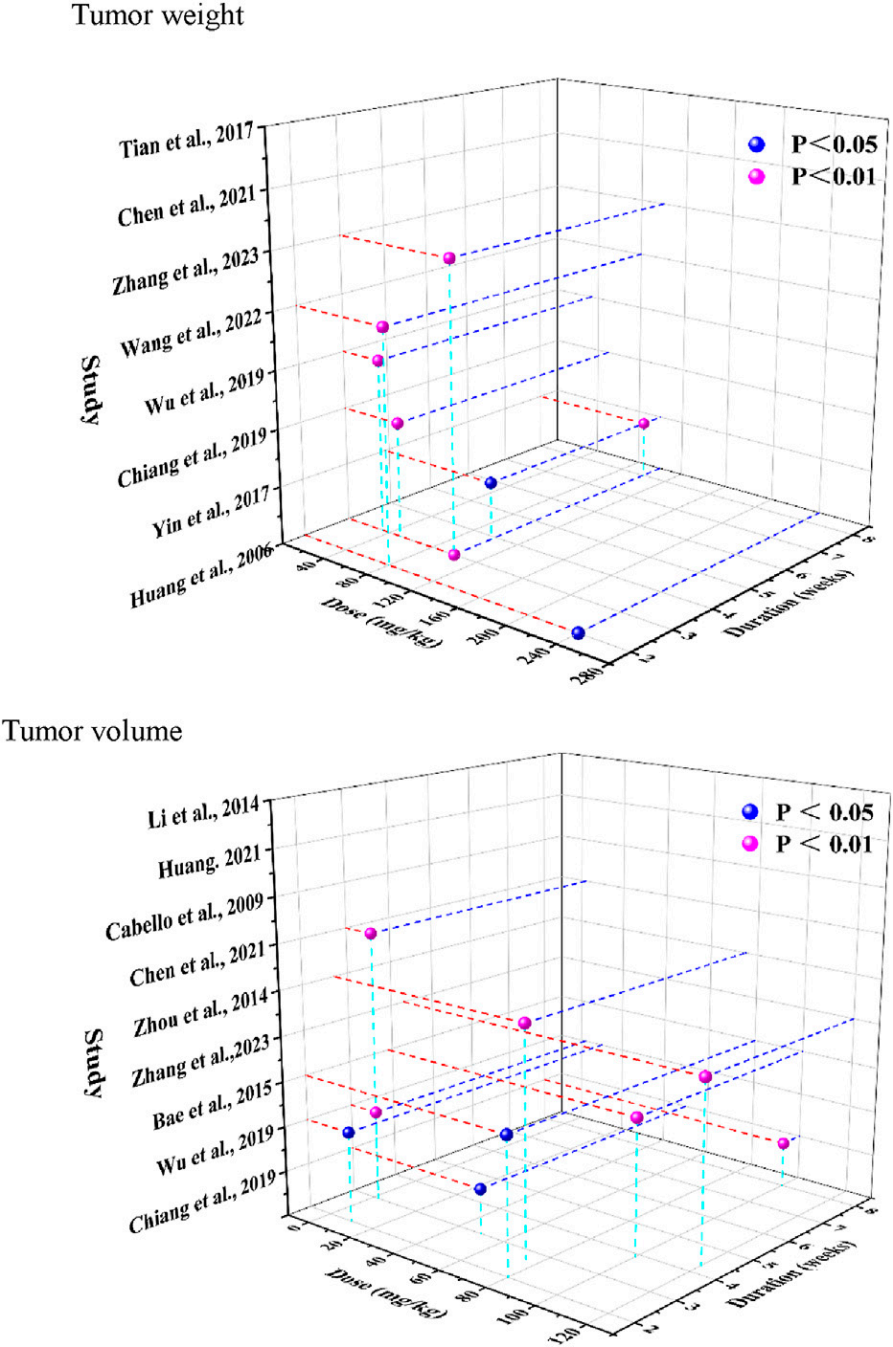
Time-dose interval analysis scatter plot for tumor weight, tumor volume.

Time- and dose-dependent patterns for tumor weight and tumor volume were visualized in Figure 8. Significant tumor weight reductions were observed in studies using CA doses ranging from 50 to 240 mg/kg, administered over 2–8 weeks (*P* < 0.05). Tumor volume reduction was evident across a broader dose range (2–120 mg/kg), with consistent findings reported throughout treatment durations of 2–8 weeks. Notably, Zhang et al. (2023) (Zhang et al., 2023) reported reductions for both tumor weight and volume (*P* < 0.01) following administration of 80 mg/kg CA over a 2-week treatment period.

### 3.8 Protein and molecular Pathways

**FIGURE 9:**
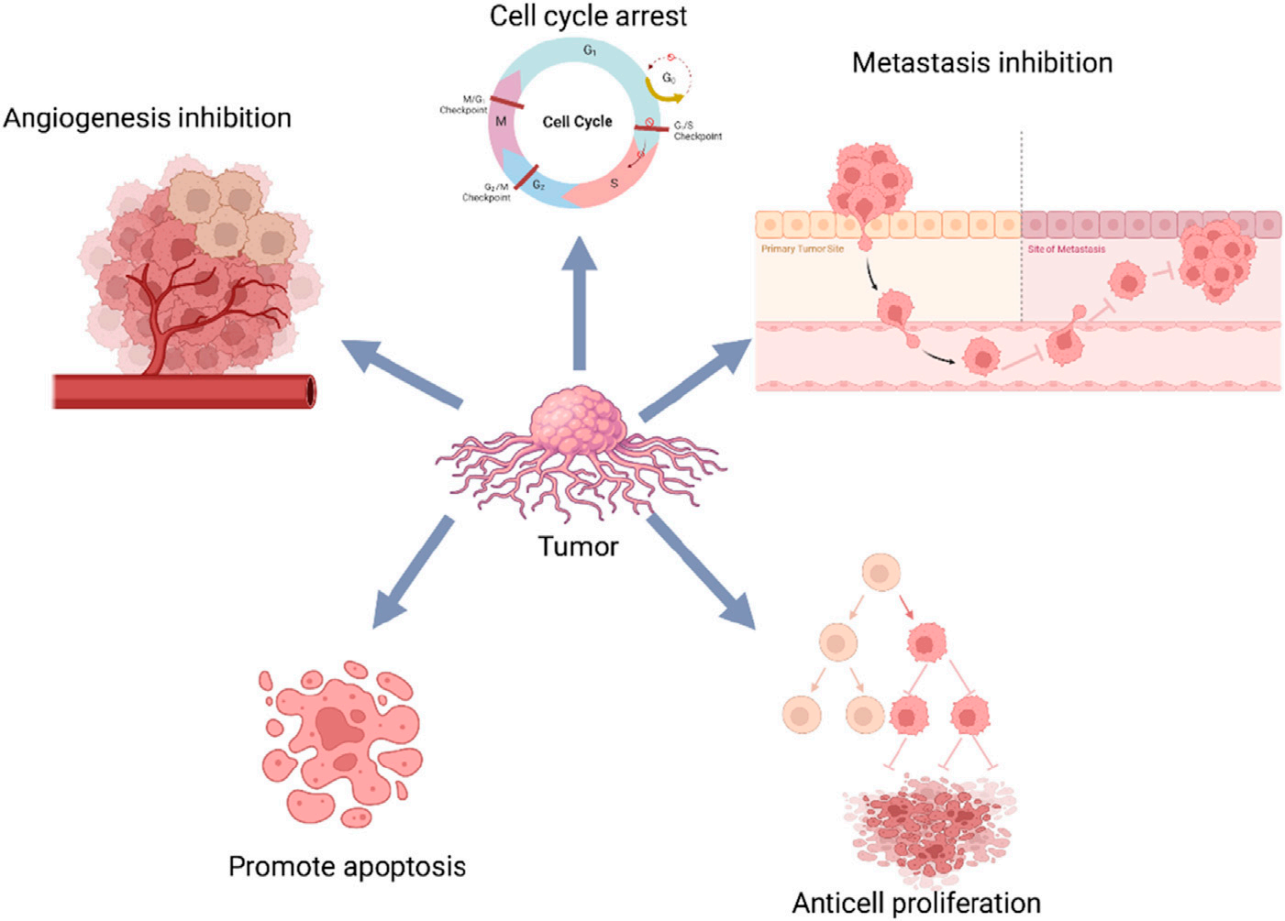
Mechanism of CA action on cancer.

As summarized in Table 6 and illustrated in Figure 9, several included studies reported cellular and molecular changes following CA administration in animal cancer models. These findings suggest that CA exposure is associated with alterations in multiple signaling pathways and regulatory proteins related to tumor progression.

**TABLE 6:** Summary of the cinnamaldehyde targeted molecular pathways and proteins in studies.

study	Cancer	Molecular pathway	Proteins	Mechanism
Huang et al., 2006	Gastric cancer	NR	NR	Cell cycle arrest (S phase accumulation), Pro-apoptosis
Yin et al., 2017	Cervical cancer	PI3K/Akt/mTOR pathway	PI3K ↓	Anti-proliferation
Chiang et al., 2019	Breast cancer	PI3K/Akt/mTOR pathway	PI3K ↓, mTOR ↓, PCNA ↓	Anti-proliferation
Chu et al., 2022	Osteosarcoma	FAK signaling pathway, NF-κB pathway, EMT (Epithelial-mesenchymal transition) pathway	u-PA ↓, p-FAK Tyr397 ↓, p-FAK Ser925 ↓, NF-κB ↓, Fibronectin ↓, N-cadherin ↓	Anti-proliferation, Anti-invasion, Anti-metastasis, Anti-EMT
Wu et al., 2019	Colorectal cancer	Wnt/β-catenin pathway, PI3K/Akt pathway, HIF-1α pathway	Bax ↑, Bcl-2 ↓, Cleaved Caspase-3 ↑, Cleaved PARP1 ↑, E-cadherin ↑, N-cadherin ↓, vimentin ↓, Snail ↓, c-Myc ↓, Cyclin D1 ↓, CD133 ↑, CD44 ↑, Oct4 ↑	Anti-proliferation, Pro-apoptosis, Anti-EMT, Anti-stemness
Wang et al., 2022	Ovarian cancer	PI3K/AKT/mTOR pathway, EGF-induced EMT pathway	p-PI3K ↓, p-AKT ↓, E-cadherin ↑, N-cadherin ↓, vimentin ↓, Snail ↓, p-mTOR ↓, cleaved-PARP ↑	Anti-proliferation, Anti-metastasis, Pro-apoptosis, Anti-EMT
Bae et al., 2015	Renal cell carcinoma	mTOR pathway, HIF-1α pathway	HIF-1α ↓, VEGF ↓, p-mTOR ↓	Anti-proliferation, Anti-angiogenesis, Anti-metastasis
Imai et al., 2002	Lung cancer	NR	NR	Anti-tumor initiation, Anti-tumor promotion
Zhang et al., 2023	Colorectal cancer	PI3K/Akt pathway, MAPK signaling pathway	p-P38 ↓, p-PI3K ↓, p-Akt ↓, Bcl-2 ↓, Survivin ↓, Bax ↑, Cleaved-caspase 3 ↑, Cleaved caspase 9 ↑, Cleaved PARP ↑, cyclinD1 ↓	Anti-proliferation, Pro-apoptosis, Cell cycle arrest, Anti-metastasis
Chen et al., 2021	Non-small cell lung cancer (NSCLC)	JAK/STAT, NF-κB, RNA degradation	p-JAK ↓, p-STAT3 ↓, p-NF-κB p65 ↓, PPARγ ↓	Anti-proliferation, Pro-apoptosis, Anti-metastasis
Cabello et al., 2009	Melanoma	NF-κB signaling pathway, Oxidative stress response	PCNA ↓, HMOX1 ↑, SRXN1 ↑, TXNRD1 ↑, CDKN1A (p21) ↑, NF-κB ↓, IL-8 ↓	Anti-proliferation, Pro-apoptosis, Anti-invasion, Anti-metastasis, Pro-oxidant
Zhou et al., 2014	Melanoma	HIF-α/VEGF pathway	HIF-α ↓, VEGF ↓	Anti-angiogenesis, Anti-tumor growth
Tian et al., 2017	Non-small cell lung cancer (NSCLC)	Wnt/β-catenin pathway, hsa_circ_0043256/miR-1252/ITCH axis	hsa_circ_0043256 ↑, miR-1252 ↓, ITCH ↑, β-catenin ↓, c-Myc ↓, CCND1 ↓, Bcl-2 ↓, Bax ↑, cleaved PARP ↑	Pro-apoptosis, Anti-proliferation, Anti-tumor growth
Huang. 2021	Osteosarcoma	Wnt/β-catenin pathway, PI3K/Akt pathway	PCNA ↓, Bcl-2 ↓, Bax ↑, cleaved caspase-3 ↑, cleaved PARP ↑, Bad ↑, N-Cadherin ↓, Snail ↓, Vimentin ↓, MMP-2 ↓, MMP-7 ↓, MMP-9 ↓, β-catenin ↓, cyclin D1 ↓, c-Myc ↓, p-GSK-3β ↓, PI3K ↓, p-Akt ↓	Anticell proliferation, Anti-invasion, Anti-metastasis
Li et al., 2014	Melanoma	HIF-α and VEGF pathways	VEGF ↓, HIF-α ↓	Anticell proliferation, Anti-angiogenesis, Anti-metastasis
Gopalakrishnan et al., 2023	Prostate cancer	Androgen receptor (AR) signaling, Proteasome pathway, Oxidative stress pathway	AR ↑, PCNA ↓, BAX ↑, Caspase-3 ↑, Caspase-8 ↑	Anti-tumor initiation, Anti-tumor promotion, Anticell proliferation, Anti-angiogenesis, Anti-metastasis, Pro-apoptosis

The reported molecular effects primarily involve markers of apoptosis, cell proliferation, metastasis, and cell cycle regulation. Among these, apoptosis-related pathways are the most frequently examined. Multiple studies demonstrated that CA treatment is associated with activation of pro-apoptotic signaling-particularly via modulation of the PI3K/Akt/mTOR and NF-κB pathways. Increased expression of pro-apoptotic proteins (e.g., Bax, cleaved caspase-3) and decreased levels of anti-apoptotic proteins (e.g., Bcl-2) were commonly observed. In addition to apoptosis-related markers, CA administration was associated with reduced expression of cell proliferation proteins, including PCNA and Cyclin D1, indicating potential suppression of tumor cell replication.

Several studies also investigated metastasis-related markers, with consistent findings showing modulation of epithelial-mesenchymal transition (EMT) components. Specifically, CA treatment was associated with decreased expression of N-cadherin and increased expression of E-cadherin, suggesting a reversal of EMT and potential inhibition of metastatic behavior. Furthermore, CA exposure influenced cell cycle progression and angiogenesis-associated markers, including components of the HIF-1α/VEGF signaling pathway. Downregulation of HIF-1αand VEGF expression, along with reduced microvessel density, was observed, indicating potential anti-angiogenic effects. Collectively, these findings suggest that CA modulates multiple molecular pathways involved in tumor growth, apoptosis, metastasis, and angiogenesis in animal models. However, due to CA’s classification as a PAINS, these effects should be interpreted cautiously and validated with rigorous mechanistic studies.

## 4 Discussion

### 4.1 Biological effects and summary of evidence

This meta-analysis synthesizes the reported effects of CA across diverse animal cancer models. Analysis of primary outcomes indicates that CA administration is consistently associated with reductions in tumor volume and weight. At the molecular level, CA-treated groups exhibited downregulation of proliferation markers such as PCNA, as well as angiogenesis-related factors including angiogenesis makers including HIF, VEGF, and microvessel density. These findings suggest that CA exerts tumor-suppressive effects across a variety of experimental cancer models, including gastric, cervical, breast, colorectal, ovarian, lung, prostate, renal cell carcinomas, melanoma, and osteosarcoma.

Substantial heterogeneity was observed in several pooled outcomes, particularly in tumor weight (*I* ^*2*^ = 76%) and tumor volume (*I* ^*2*^ = 81%). Subgroup analyses identified that cancer type was the primary contributor of heterogeneity in tumor weight outcomes. CA treatment was associated with tumor reduction in models of gastric, cervical, colorectal, ovarian, and lung cancers. In contrast, no significant effect was observed in breast cancer models (SMD = −2.11, 95% CI [−4.71, 0.48], *P* = 0.11), suggesting that biological responses to CA may be tumor-type dependent.

In tumor volume analyses, dosage emerged as the key factor affecting heterogeneity. High-dose groups (≥100 mg/kg) demonstrated more consistent effects (*I* ^*2*^ = 19%) than lower doses. Treatment duration did not appear to influence outcome variability, with all duration-based subgroups exhibiting low heterogeneity (*I* ^*2*^ = 0%). Evaluation of administration routes indicated that subcutaneous injections yielded the strongest effect size (SMD = −6.83), albeit with higher heterogeneity (*I* ^*2*^ = 87%), while intraperitoneal injections produced more consistent results (SMD = −2.70, *I* ^*2*^ = 53%). Additionally, species-related differences were noted, with mouse models generally showing stronger responses to CA than rat models, further contributing to experimental variability.

Notably, subgroup analysis of melanoma models revealed a large pooled effect size (SMD = −6.22), although the result did not reach statistical significance (*P* = 0.09). The high heterogeneity among melanoma studies (*I* ^*2*^ = 94%; *P* < 0.00001) may be attributed to variations in administration route (intragastric, intraperitoneal, subcutaneous), dosage (2–120 mg/kg), and small sample sizes. Given the clinical challenges associated with melanoma treatment, these preliminary findings suggest potential relevance for further exploration in well-controlled studies.

Overall, these findings highlight both the biological activity and the substantial variability of CA’s effects in experimental cancer models. Differences in tumor type, dosage, administration method, and animal species all appear to influence study outcomes. These observations underscore the need for rigorous standardization and mechanistic validation in future investigations.

### 4.2 Safety considerations and Therapeutic window

Our safety analysis revealed a modest decrease in body weight in CA-treated groups (SMD = −0.65, *P* = 0.003), which may indicate metabolic effects or mild toxicity that underscores the need for further investigation into CA’s systemic impact. Of greater concern is the inconsistency in reported survival outcomes (*I* ^*2*^ = 69%, *P* = 0.006), with pooled analysis showing no significant survival difference between treated and control groups (RR = 1.03, 95% CI [0.85, 1.25], *P* = 0.77). The absence of a survival benefit, despite reductions in tumor burden, raises important questions about the overall biological relevance and durability of CA’s tumor-suppressive effects.

Dosing across included studies ranged from 2 to 240 mg/kg in animals, which corresponds to approximately 0.16–19.5 mg/kg in humans when converted using FDA-recommended body surface area correction factors (Reagan-Shaw et al., 2008). According to Adams et al. (2004) (Adams et al., 2004), the estimated average daily dietary intake of CA in the U.S. population is approximately 59.3 mg. The considerably higher doses required to elicit tumor-suppressive effects in animal models far exceed typical dietary exposures, emphasizing a

significant translational gap. This discrepancy raises important concerns about the potential for toxicity if CA were to be administered at pharmacologically active levels in a therapeutic context. Notably, the long-term safety profile of CA remains poorly characterized. Most studies did not include comprehensive toxicological assessments, such as evaluations of hepatic and renal function, hematologic indices, or histopathological analysis of major organs. While high-dose CA appeared to yield greater reductions in tumor volume, no systematic assessment of dose dependent toxicity was conducted. This lack of safety data limits any conclusions regarding the therapeutic window of CA.

In the absence of robust toxicological evaluation, it is premature to consider CA for further therapeutic development. Future studies must include standardized safety endpoints to determine whether the observed tumor-suppressive effects can be achieved without compromising organismal health.

### 4.3 Limitations and considerations

While this review systematically evaluates the reported biological effects of CA in animal cancer models, several important limitations must be acknowledged. First, substantial heterogeneity was observed across studies, even after stratified subgroup analyses by species, tumor model, cancer type, administration route, dosage, and treatment duration. In some cases, such as the melanoma subgroup (*I* ^*2*^ = 94%), the heterogeneity remained unresolved despite clear trends in effect direction. Several pooled analyses demonstrated large effect sizes that did not reach statistical significance, largely due to wide confidence intervals and high inter-study variability. For subgroups comprising a single study-such as those involving rat models or less commonly studied cancer types-heterogeneity could not be statistically assessed, further limiting interpretability. These statistical issues highlight the preliminary and exploratory nature of the current evidence base.

In terms of methodological quality, the overall risk of bias was high across the included studies, as assessed by the SYRCLE risk of bias tool. Key design safeguards-such as randomization, blinding, allocation concealment, and proper outcome assessment protocols-were frequently absent or inadequately reported. These omissions introduce a considerable risk of systematic bias and compromise the internal validity of reported findings. In addition, publication bias cannot be ruled out, as studies with null or negative results may be underrepresented in the published literature. Other limitations include possible language bias due to the exclusion of non-English articles and potential measurement bias from indirect data extraction (e.g., from graphs rather than raw values), which may reduce data accuracy.

The distinction between naturally extracted and synthetically produced CA also warrants consideration. While both forms are chemically identical, naturally derived CA may contain trace compounds from plant matrices that could influence biological activity. Furthermore, limited reporting on CA purity and analytical validation in many studies complicates the interpretation of dose-response relationships and mechanism-specific effects.

One of the most critical limitations is the lack of appropriate chemical controls. Very few studies employed structurally similar but non-reactive analogs—such as cinnamic acid or

hydrocinnamaldehyde-which are essential for distinguishing specific biological activity from effects due to CA’s reactive aldehyde group. Without such controls, it remains unclear whether the observed outcomes reflect targeted molecular actions or are artifacts of nonspecific chemical reactivity.

Overall, these limitations reinforce the need for more rigorously designed, better-controlled, and mechanistically validated studies to clarify the biological relevance and specificity of CA’s effects in cancer models.

### 4.4 Cinnamaldehyde as a Pan-assay interference compound: challenges in distinguishing specific effects from chemical artifacts

A critical consideration in interpreting the reported anti-tumor effects of CA is its chemical classification as a PAINS. This designation reflects its intrinsic electrophilic reactivity, which fundamentally challenges the biological plausibility of its purported multi-target activity in cancer models.

CA readily forms covalent adducts with nucleophilic residues-particularly cysteine thiols and lysine amines-on proteins such as Keap1 and IKKβ, indiscriminately affecting redox-sensitive signaling pathways including NF-κB and Nrf2. This chemical behavior likely accounts for the frequently observed downregulation of HIF-1α and VEGF, which are highly sensitive to oxidative stress. Such changes may reflect global redox disruption rather than true anti-angiogenic or pathway-specific effects.

The reported modulation of diverse pathways-PI3K/Akt/mTOR, MAPK, NF-κB, and numerous regulatory proteins including PCNA, HIF, and VEGF-is more consistent with a generalized cellular stress response than with targeted pharmacological action. Observed effects on apoptosis-related markers, such as Bax and cleaved caspases, may similarly stem from non-specific oxidative or proteotoxic stress rather than mechanistically selective induction of cell death pathways. Importantly, none of the included studies employed non-electrophilic structural analogs (e.g., dihydrocinnamaldehyde) to control for PAINS-associated artifacts. The absence of such controls prevents differentiation between specific pharmacological mechanisms and non-specific chemical reactivity. This represents a critical gap in the current literature and reflects a broader challenge in evaluating reactive natural products.

Additionally, the doses required to elicit anti-tumor effects in animal studies (50–240 mg/kg) translate to human-equivalent doses (HED) of approximately 4–19.5 mg/kg, which greatly exceed typical dietary exposures (∼59.3 mg/day). This dose disparity raises concerns about toxicity and physiological relevance. Although reductions in tumor volume were observed, these effects were accompanied by a modest but significant decrease in body weight, suggesting potential systemic toxicity. More critically, the lack of survival benefit (RR = 1.03; 95%CI [0.85, 1.25]; *P* = 0.77) underscores a disconnect between tumor burden reduction and meaningful biological outcome.

Taken together, the available evidence does not support CA as a selective anti-cancer agent. Instead, the observed effects likely represent the biological consequences of administering a reactive electrophilic compound to living systems. Without rigorous mechanistic studies-including the use of non-reactive analogs, proteomic quantification of covalent adducts, comprehensive toxicological profiling, and pathway-specific biomarkers-CA’s anti-tumor effects remain indistinguishable from chemical artifacts.

Future research should prioritize methodological improvements that allow clear distinction between genuine pharmacological action and non-specific chemical interference. Until such studies are undertaken, CA should not be considered a viable candidate for therapeutic development, and its biological effects should be interpreted with appropriate caution.

## 5 Conclusion

This systematic review and meta-analysis demonstrates that CA administration is consistently associated with reduced tumor volume and tumor weight in a range of animal cancer models. However, the interpretation of these findings is significantly limited by multiple critical factors that challenge their biological and translational relevance.

Foremost among these is CA’s classification as a PAINS, owing to its electrophilic α,β-unsaturated aldehyde structure. This chemical feature enables widespread, non-specific protein adduction, particularly targeting nucleophilic residues in redox sensitive signaling proteins. None of the included studies utilized non-reactive structural analogs to control for these potential artifacts, making it impossible to distinguish between specific pharmacological activity and general chemical reactivity.

Furthermore, although CA treatment was associated with measurable reductions in tumor size, no survival benefit was observed, underscoring a disconnect between tumor suppression and clinically relevant outcomes. This finding raises the possibility that tumor shrinkage may be the result of generalized cytotoxic stress rather than mechanistically targeted anti-cancer activity.

In addition, the methodological quality of the included studies was generally low, with frequent deficiencies in randomization, blinding, allocation concealment, and outcome reporting. Significant inter-study heterogeneity, particularly across tumor types and administration protocols, further limits the interpretability and reproducibility of the results.

Taken together, the available evidence does not support the conclusion that CA possesses

specific anti-cancer properties suitable for clinical translation. Instead, these findings highlight the importance of applying rigorous methodological frameworks when evaluating natural compounds with reactive chemical structures.

Future studies should adopt a more robust experimental design, including: (1) The use of non-reactive analogs to control for PAINS-associated effects; (2) Comprehensive toxicological assessments across relevant dose ranges; (3) Proteomic analyses to directly quantify protein adduct formation; (4) And standardized, bias-controlled protocols with adequate sample sizes.

This critical assessment of CA illustrates the broader challenges inherent in evaluating reactive natural products in preclinical cancer research. It underscores the necessity of distinguishing true biological specificity from non-specific chemical stress, ensuring that laboratory observations are interpreted with appropriate chemical and mechanistic context before being considered for therapeutic development.

## Abbreviations

CA, Cinnamaldehyde; EMT, Epithelial-Mesenchymal Transition; MAPK, Mitogen-activated protein kinase; Nrf2, Nuclear factor erythroid 2-related factor 2; NF-κB, Nuclear Factor Kappa B; HIF, Hypoxia-Inducible Factor; VEGF, Vascular Endothelial Growth Factor; PCNA, Proliferating Cell Nuclear Antigen; TME, Tumor Microenvironment; SYRCLE, Systematic Review Centre for Laboratory Animal Experimentation; SMD, Standardized Mean Difference; CI, Confidence Interval; PAINS, pan-assay interference compound.
