# Supplementary material for: Biological effects of cinnamaldehyde in animal cancer models: a systematic review and meta-analysis
Source: Front Pharmacol. 2025 May 16;16:1557088. doi: 10.3389/fphar.2025.1557088 (PMC12122501; doi:10.3389/fphar.2025.1557088)
Supplement: Supplementary file 2 [file Table1.docx]

Search strategies

| Literature databases | Search items | Items found |
| --- | --- | --- |
| (1) PubMed | (cinnamaldehyde[MeSH Terms] OR cinnamaldehyde[Title/Abstract] OR "cinnamic aldehyde"[Title/Abstract])  AND  (neoplasms[MeSH Terms] OR cancer[Title/Abstract] OR tumor[Title/Abstract] OR tumour[Title/Abstract] OR neoplasm*[Title/Abstract])  AND  (animals, laboratory[MeSH Terms] OR animal experimentation[MeSH Terms] OR models, animal[MeSH Terms] OR mice[MeSH Terms] OR rats[MeSH Terms] OR "animal model*"[Title/Abstract] OR "animal experiment*"[Title/Abstract] OR mouse[Title/Abstract] OR mice[Title/Abstract] OR rat[Title/Abstract] OR rats[Title/Abstract] OR "in vivo"[Title/Abstract] OR "preclinical"[Title/Abstract]) | 160 |
| (2)Science Direct | #1  Title, abstract, keywords: cinnamaldehyde OR "cinnamic aldehyde"  #2  cancer OR tumor OR tumour OR neoplasm  #3  "animal model" OR "animal experiment" OR mouse OR mice OR rat OR rats OR "in vivo" OR preclinical  #1 AND #2 #3 | 50 |
| (3)Web of science | ALL=( (cinnamaldehyde OR "cinnamic aldehyde") AND (neoplasm* OR cancer* OR tumor* OR tumour*) AND ("animal model*" OR "animal experiment*" OR mouse OR mice OR rat OR rats OR "in vivo" OR preclinical OR rodent*) )  Timespan: All years. Indexes: SCI-EXPANDED. | 177 |
| (4)Google Scholar | (“Acute Ischemic Stroke” OR “Ischemic Strokes” OR “Cryptogenic Ischemic Stroke” OR “Cryptogenic Embolism Stroke” OR “Wake-up Stroke” OR “Cryptogenic Embolism Strokes” ) AND (“Berberine” OR “Berberine Alkaloids” OR “Huangliansu”) | 156 |
| (5)Embase | ('cinnamaldehyde'/exp OR cinnamaldehyde:ti,ab OR 'cinnamic aldehyde':ti,ab,kw)  AND  ('neoplasm'/exp OR cancer:ti,ab,kw OR tumor:ti,ab,kw OR tumour:ti,ab,kw OR neoplasm*:ti,ab,kw)  AND  ('animal experiment'/exp OR 'animal model'/exp OR 'laboratory animal'/exp OR mouse:ti,ab,kw OR mice:ti,ab,kw OR rat:ti,ab,kw OR rats:ti,ab,kw OR 'in vivo':ti,ab,kw OR preclinical:ti,ab,kw) | 252 |
| (6)China National Knowledge Infrastructure Database | (SU='肉桂醛' OR KY='肉桂醛' OR AB='肉桂醛')  AND  (SU='癌症' OR SU='肿瘤' OR SU='恶性肿瘤' OR KY='癌症' OR KY='肿瘤' OR KY='恶性肿瘤' OR TI='癌症' OR TI='肿瘤' OR TI='恶性肿瘤' OR AB='癌症' OR AB='肿瘤' OR AB='恶性肿瘤')  AND  (SU='动物实验' OR SU='动物模型' OR SU='小鼠' OR SU='大鼠' OR SU='体内实验' OR SU='临床前研究' OR KY='动物实验' OR KY='动物模型' OR KY='小鼠' OR KY='大鼠' OR KY='体内实验' OR KY='临床前研究' OR TI='动物实验' OR TI='动物模型' OR TI='小鼠' OR TI='大鼠' OR TI='体内实验' OR TI='临床前研究' OR AB='动物实验' OR AB='动物模型' OR AB='小鼠' OR AB='大鼠' OR AB='体内实验' OR AB='临床前研究' OR FT='动物实验' OR FT='动物模型' OR FT='小鼠' OR FT='大鼠' OR FT='体内实验' OR FT='临床前研究') | 164 |
| (7)Wan Fang database Search strategy | (主题词=肉桂醛 OR 关键词=肉桂醛 OR 题名=肉桂醛 OR 摘要=肉桂醛)  AND  (主题词=(癌症 OR 肿瘤 OR 恶性肿瘤) OR 关键词=(癌症 OR 肿瘤 OR 恶性肿瘤) OR 题名=(癌症 OR 肿瘤 OR 恶性肿瘤) OR 摘要=(癌症 OR 肿瘤 OR 恶性肿瘤))  AND  (主题词=(动物实验 OR 动物模型 OR 小鼠 OR 大鼠 OR 体内实验 OR 临床前研究) OR  关键词=(动物实验 OR 动物模型 OR 小鼠 OR 大鼠 OR 体内实验 OR 临床前研究) OR  题名=(动物实验 OR 动物模型 OR 小鼠 OR 大鼠 OR 体内实验 OR 临床前研究) OR  摘要=(动物实验 OR 动物模型 OR 小鼠 OR 大鼠 OR 体内实验 OR 临床前研究)) | 90 |
| (8)Chongqing VIP Chinese Science and Technology Periodical Database (VIP) | (M=肉桂醛 OR K=肉桂醛 OR T=肉桂醛 OR R=肉桂醛)  AND  (U=(癌症 OR 肿瘤 OR 恶性肿瘤) OR M=(癌症 OR 肿瘤 OR 恶性肿瘤) OR K=(癌症 OR 肿瘤 OR 恶性肿瘤) OR T=(癌症 OR 肿瘤 OR 恶性肿瘤) OR R=(癌症 OR 肿瘤 OR 恶性肿瘤))  AND  (U=(动物实验 OR 动物模型 OR 小鼠 OR 大鼠 OR 体内实验 OR 临床前研究) OR  M=(动物实验 OR 动物模型 OR 小鼠 OR 大鼠 OR 体内实验 OR 临床前研究) OR  K=(动物实验 OR 动物模型 OR 小鼠 OR 大鼠 OR 体内实验 OR 临床前研究) OR  T=(动物实验 OR 动物模型 OR 小鼠 OR 大鼠 OR 体内实验 OR 临床前研究) OR  R=(动物实验 OR 动物模型 OR 小鼠 OR 大鼠 OR 体内实验 OR 临床前研究)) | 11 |
| Overall |  | 1060 |
